# Short-Term Decreasing and Increasing Dietary BCAA Have Similar, but Not Identical Effects on Lipid and Glucose Metabolism in Lean Mice

**DOI:** 10.3390/ijms24065401

**Published:** 2023-03-11

**Authors:** Yuchen Sun, Bo Sun, Zhishen Wang, Yinfeng Lv, Qingquan Ma

**Affiliations:** College of Animal Science and Technology, Northeast Agricultural University, Harbin 150030, China

**Keywords:** branched-chain amino acid, diet-induced obesity, mouse, lipid metabolism, glucose metabolism

## Abstract

Branched-chain amino acids (BCAA) showed multiple functions in glycolipid metabolism and protein synthesis. However, the impacts on the metabolic health of low or high dietary BCAA remain controversial due to the various experimental conditions. Gradient levels of BCAA were supplemented in lean mice for four weeks: 0BCAA (without BCAA), 1/2BCAA (half BCAA), 1BCAA (regular BCAA), and 2BCAA (double BCAA). The results showed that the diet without BCAA caused energy metabolic disorders, immune defects, weight loss, hyperinsulinemia, and hyperleptinemia. 1/2BCAA and 2BCAA diets reduced body fat percentage, but 1/2 BCAA also decreased muscle mass. 1/2BCAA and 2BCAA groups improved lipid and glucose metabolism by affecting metabolic genes. Meanwhile, significant differences between low and high dietary BCAA were observed. The results of this study provide evidence and reference for the controversy about dietary BCAA levels, which indicates that the main difference between low and high BCAA dietary levels may present in the longer term.

## 1. Introduction

Obesity is a growing threat to human health worldwide, leading to severe metabolic complications, including diabetes mellitus, insulin and leptin resistance, and other metabolic disorders. A primary cause of obesity is improper food consumption, specifically of diets with high fat (HFD) [1]. Therefore, dietary intervention is an effective means to prohibit obesity.

Amino acids (AAs), as critical nutritive substances in diets, can not only play a vital role in nutrition, but also exhibit various functions in immuno-stimulation, metabolism, or physiology. The proposal and application of the concept of functional amino acids have been proven effective in mitigating diseases impairing animal health [2]. For instance, dietary threonine supplementation notably reduces fat mass, improves insulin resistance, and facilitates lipid metabolic health in obese mice [3]. The increased dietary intake of aromatic amino acids (phenylalanine, tryptophan, and tyrosine) improved lipid metabolism and insulin tolerance by stimulating bile acid synthesis [4]. Moreover, leucine (Leu), isoleucine (Ile), and valine (Val), which are called branched-chain amino acids (BCAAs), are always research hot spots because of their association with type 2 diabetes, insulin resistance, and obesity [5,6]. Substantial evidence indicates that dietary BCAA supplementation can improve muscle development, insulin sensitivity, and lipid accumulation in high-fat, diet-induced, obese (DIO) mice [7,8,9].

However, the notion that BCAA supplementation could be positive for lipid metabolism or insulin function remains controversial [10]. Reduced consumption of BCAAs effectively antagonized obesity and insulin resistance [11]. A recent report showed that the adverse metabolic effects in lean mice were induced by Ile and Val, not Leu [12]. Yet, our previous study demonstrated that elevated dietary Leu, rather than Ile and Val, worsen the lipid metabolism in lean mice [13]. These results are affected by various experimental conditions, such as the mouse model, diet composition, or the duration of the test. Thus, more evidence is needed to verify the effects of dietary BCAA regulation under different conditions.

In this study, we explore the effect of four different dietary BCAA levels (without, low, regular, and high BCAA levels included) by purified diet on lipid and glucose metabolism in normal lean mice under the same conditions. The total BCAA deprivation experiment was designed to indirectly investigate the role of BCAAs in body development, which was also a preliminary study for the following individual BCAA-removed trials.

## 2. Results

### 2.1. Different Dietary BCAA Levels Influenced the Ratio of Muscle and WAT in Lean Mice

**Figure 1:**
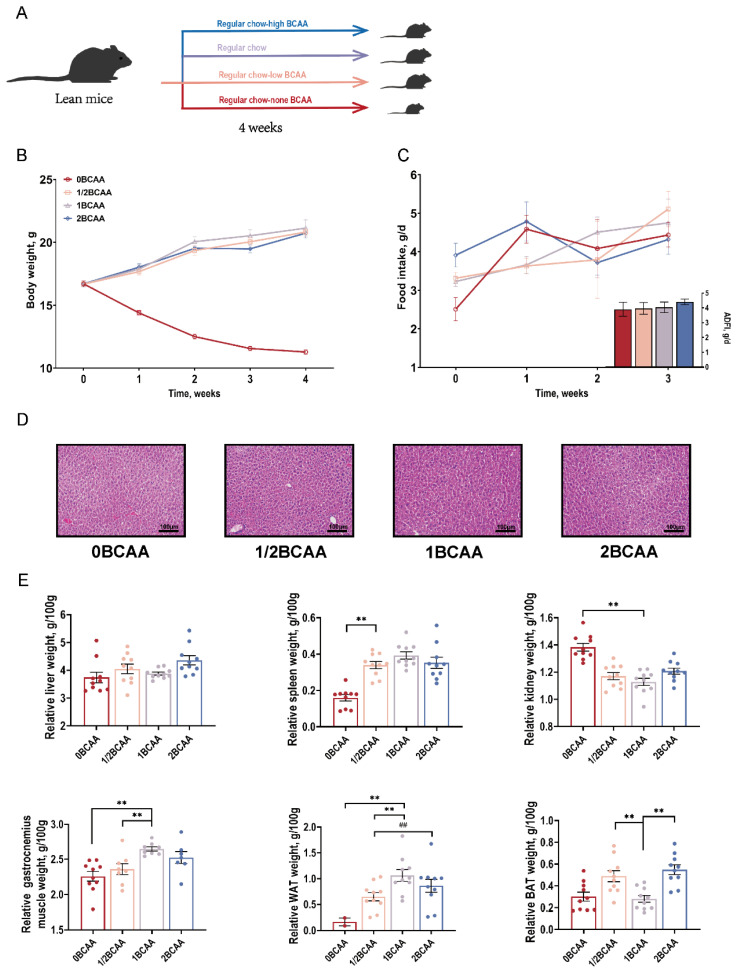
Effects of different dietary BCAA levels on the growth performance and organ development of lean mice. (**A**): Experimental design; (**B**): Body weight curve; (**C**): Feed intake for 4 weeks; (**D**): Representative H&E staining images of the liver; (**E**): Relative organ weight. Values are presented as the means ± SEM (*n* = 15 of growth performance and ADFI, *n* = 8 of relative organ weights). ADFI: Average daily feed intake; WAT: White adipose tissue; BAT: Brown adipose tissue. ** *p* < 0.01 versus 1BCAA group. ## *p* < 0.01 in 2BCAA group versus 1/2BCAA group.

Lean mice were provided diets with four different BCAA levels for four weeks (Figure 1A). There were no significant differences in body weight among the 1/2BCAA, 1BCAA, and 2BCAA groups. Completely removing dietary BCAA significantly reduced body weight during the experimental period (Figure 1B). However, no marked difference in the average daily feed intake among the four groups was observed (Figure 1C). At the end of the fourth week, liver histopathological observation and relative liver weight showed no significant difference among experiment groups with different dietary BCAA levels (Figure 1D,E). The diet without BCAA significantly decreased the relative organ weight of the spleen, gastrocnemius muscle, and WAT, and increased the relative kidney weight. We also observed a lower relative weight of WAT in the 1/2BCAA group compared with the 1BCAA and 2BCAA groups, a lower relative weight of gastrocnemius muscle and a higher relative weight of BAT in 1/2BCAA and 2BCAA groups compared with 1BCAA group (Figure 1E). Half or doubled dietary BCAA levels showed the same trend in WAT and BAT.

### 2.2. Complete Deprivation of Dietary BCAA Impaired Glucose Homeostasis

**Figure 2:**
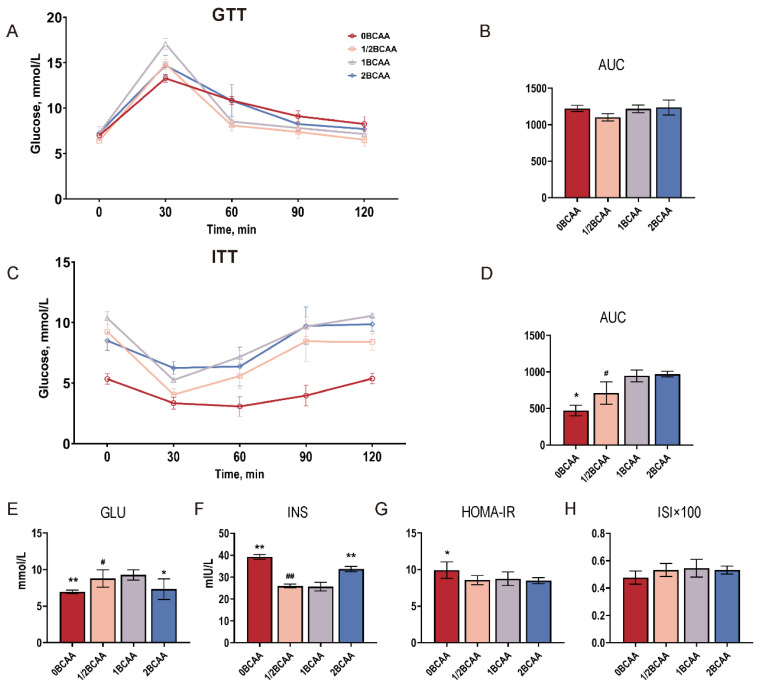
Effects of different dietary BCAA levels on glucose homeostasis in lean mice. (**A**): GTT curve; (**B**): GTT AUC; (**C**): ITT curve; (**D**): ITT AUC; (**E**): Serum GLU level; (**F**): Serum INS level; (**G**): HOMA-IR; (**H**): ISI index. Values are presented as the means ± SEM (*n* = 8). GTT: Glucose tolerance test; ITT: Insulin tolerance test; AUC: Areas under the curves; GLU: Glucose; INS: Insulin; HOMA-IR: Homeostasis model assessment of insulin resistance; ISI: Improved insulin sensitivity index. Values are presented as the means ± SEM (*n* = 8). * *p* < 0.05, ** *p* < 0.01 versus 1BCAA group. # *p* < 0.05, ## *p* < 0.01 in 2BCAA group versus 1/2BCAA group.

Different levels of BCAA had no significant effect on glucose homeostasis in lean mice (Figure 2A,B). However, non-BCAA and low-BCAA diets decreased the area under the curve (AUC) of ITT (Figure 2C,D). BCAA removal reduced the GLU level and increased the serum insulin level, thus inducing a poor performance in HOMA-IR (Figure 2E–G). The 2BCAA group had a lower GLU level and higher insulin level in the serum compared with the 1/2BCAA and 1BCAA groups, which did not affect the HOMA-IR and ISI (Figure 2E–H).

### 2.3. Different Dietary BCAA Levels Altered the Serum Indexes in Lean Mice

**Figure 3:**
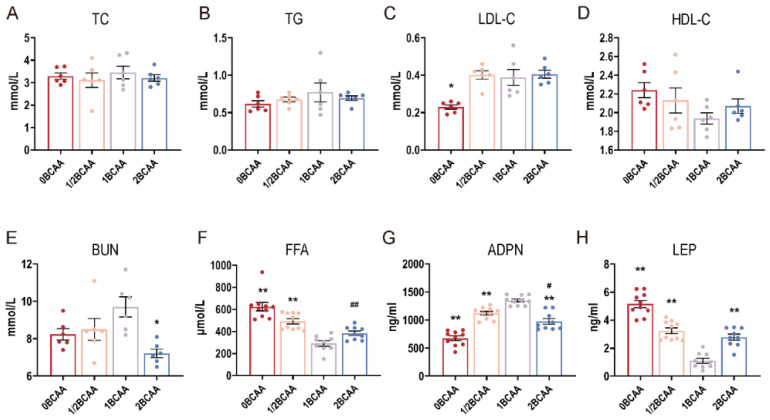
Effects of different dietary BCAA levels on serum biochemical indices in lean mice. (**A**): Total cholesterol; (**B**): Total triglycerides; (**C**): Low-density lipoprotein cholesterol; (**D**): High-density lipoprotein cholesterol; (**E**): Urea nitrogen; (**F**): Free fatty acid; (**G**): Adiponectin; (**H**): Leptin. Values are presented as the means ± SEM. (**A**–**E**): *n* = 6; (**F**–**H**): *n* = 10. TC: Total cholesterol; TG: Total triglycerides; LDL-C: Low-density lipoprotein cholesterol; HDL-C: High-density lipoprotein cholesterol; BUN: Urea nitrogen; FFA: Free fatty acid; ADPN: Adiponectin; LEP: Leptin. * *p* < 0.05, ** *p* < 0.01 versus 1BCAA group. # *p* < 0.05, ## *p* < 0.01 in 2BCAA group versus 1/2BCAA group.

Different dietary BCAA levels had no significant influence on serum TC, TG, and HDL-C in lean mice (Figure 3). Mice fed with a diet without BCAA had a lower LDL-C and ADPN, and higher FFA and LEP in the serum compared with the control group. Half the dietary BCAA level elevated FFA, ADPN, and LEP serum levels. Compared with the 1BCAA group, a 2BCAA diet decreased BUN and ADPN levels and increased LEP level. Besides, the FFA and ADPN levels in the 1/2BCAA group were superior to those in the 2BCAA group.

### 2.4. Different Dietary BCAA Levels Have Different Effects on Metabolic Genes

To further determine the variations in transcriptome among groups with different dietary BCAA levels, transcriptome analyses were performed. The volcano plot showed differentially expressed genes in the liver of mice (Figure S1A). As shown in the violin plot, there was no distinct difference in the abundance of differentially expressed genes (DEGs) in different treatments (Figure S1B). The heatmap and hierarchical clusters analysis (Figure S1C) highlighted significant differences in the expression levels of the top 50 DEGs among the four groups. The counts of DEGs in 0BCAA vs. 1BCAA (455 up-regulated genes and 1127 down-regulated genes), 1/2BCAA vs. 1BCAA (175 up-regulated genes and 188 down-regulated genes), 2BCAA vs. 1BCAA (327 up-regulated genes and 107 down-regulated genes), and 1/2BCAA vs. 2BCAA (63 up-regulated genes and 203 down-regulated genes) were demonstrated (Figure S1D).

**Figure 4:**
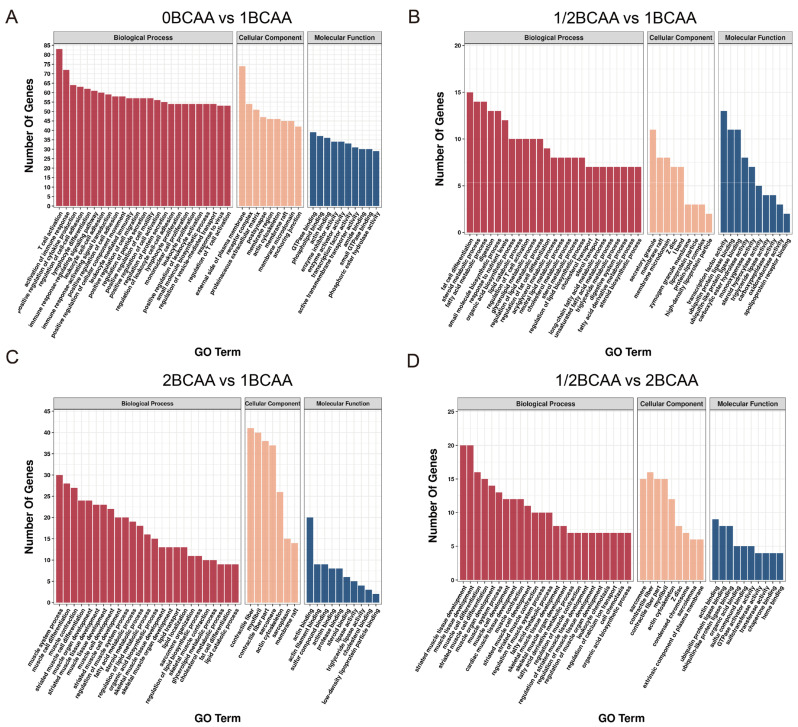
GO enrichment analysis of differentially expressed genes in four groups. (**A**): 0BCAA vs. 1BCAA; (**B**): 1/2BCAA vs. 1BCAA; (**C**): 2BCAA vs. 1BCAA; (**D**): 1/2BCAA vs. 2BCAA. GO: Gene ontology; BP: Biological processes; CC: Cellular components; MF: Molecular functions.

GO functional enrichment analysis was performed to investigate the potential functions of the DEGs (Figure 4). Enriched GO terms targeted by DEGs between 0BCAA and 1BCAA were mainly related to an immune function and inflammation. Insulin secretion and response to insulin were also significantly enriched. Besides, terms related to lipid metabolism, carbohydrate metabolism, and muscle development were markedly enriched among 1/2BCAA, 1BCAA, and 2BCAA groups: “fat cell differentiation”, “fatty acid metabolic process”, “lipid catabolic process”, “cholesterol transport”, “skeletal system development”, “muscle tissue development”, “glycogen (starch) synthase activity”, and so on.

**Figure 5:**
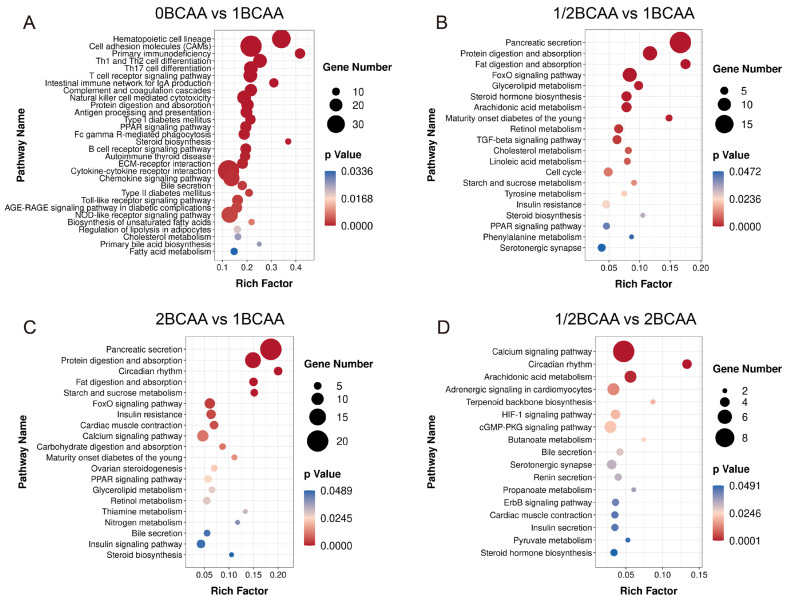
KEGG enrichment analysis of differentially expressed genes in four groups. (**A**): 0BCAA vs. 1BCAA; (**B**): 1/2BCAA vs. 1BCAA; (**C**): 2BCAA vs. 1BCAA; (**D**): 1/2BCAA vs. 2BCAA; KEGG: Kyoto encyclopedia of genes pathway.

KEGG enrichment analysis revealed that dietary BCAA levels could influence the pathways involved in lipid, carbohydrate, protein, and insulin metabolism (Figure 5), including “fatty acid metabolism”, “fat digestion and absorption”, “cholesterol metabolism”, “bile secretion”, “starch and sucrose metabolism”, “carbohydrate digestion and absorption”, “protein digestion and absorption”, “PPAR signaling pathway”, “pancreatic secretion”, “insulin signaling pathway”, and “insulin resistance”.

**Figure 6:**
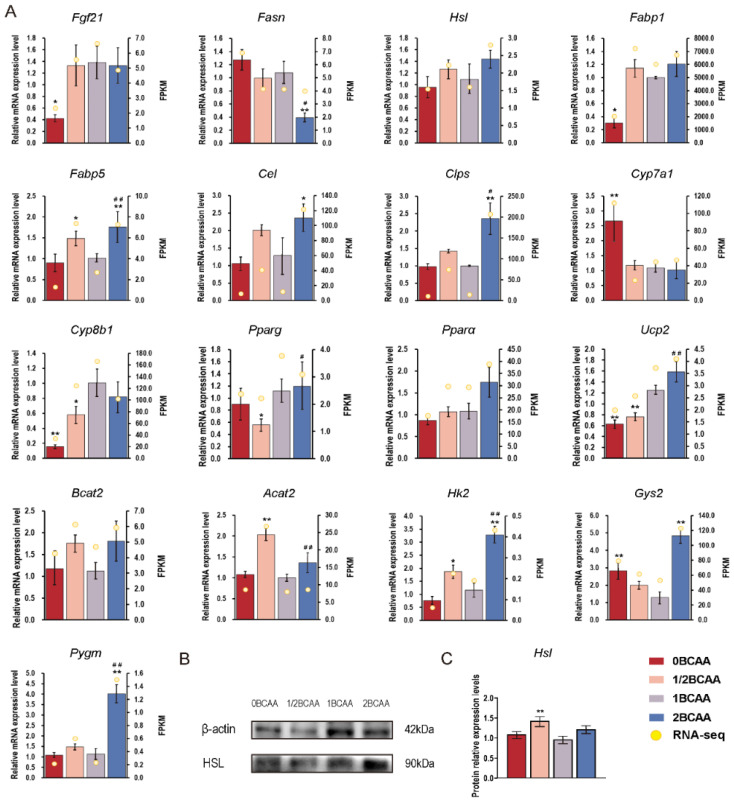
Effects of different dietary BCAA levels on genes related to lipid and glucose metabolism in lean mice. (**A**): RT-PCR validation of transcriptomic analysis; (**B**): Western blot analysis of *HSL*; (**C**): Relative protein expression level. Values are presented as the means ± SEM (*n* = 8). *Fgf21*: Fibroblast growth factor 21; *Fasn*: Fatty acid synthase; *Hsl*: Hormone sensitive lipase; *Fabp1*: Fatty acid-binding protein 1; *Fabp5*: Fatty acid-binding protein 5; *Cel*: Carboxyl ester lipase; *Clps*: Colipase; *Cyp7a1*: Cytochrome P450 family 7 subfamily A member 1; *Cyp8b1*: Cytochrome P450 family 8 subfamily B member 1; *Pparg*: Peroxisome proliferator-activated receptor gamma; *Pparα*: Peroxisome proliferator-activated receptor alpha; *Ucp2*: Uncoupling protein 2; *Bcat2*: Branched-chain aminotransferase 2; *Acat2*: Acetyl-Coenzyme A acetyltransferase 2; *Hk2*: Hexokinase 2; *Gys2*: Glycogen synthase 2; *Pygm*: Muscle glycogen phosphorylase; FPKM: Fragments Per Kilobase of exon model per Million mapped fragments. * *p* < 0.05, ** *p* < 0.01 versus 1BCAA group. # *p* < 0.05, ## *p* < 0.01 in 2BCAA group versus 1/2BCAA group.

Seventeen genes related to lipid and glucose metabolism affected by different BCAA levels were selected for validation through real-time PCR (Table 1). The results of real-time PCR validation are shown in Figure 6A, consistent with the transcriptomic analyses, which proved that the results from transcriptomic analyses can be trusted. The secondary verification was performed by western blot, and the relative protein expression of HSL agreed with the data in the study (Figure 6B,C).

## 3. Discussion

Our previous studies demonstrated that BCAAs had different impacts on lipid metabolism in mice under different conditions (lean or obese) [9,13], which was also proved by other researchers [12]. However, the results from these trials always had more or less differences. The never-ceased controversies highlight the importance of test conditions. In this study, the effects of 1/2BCAA and 2BCAA levels on lipid and glucose metabolism in lean mice are considered favorable, but achieved through different mechanisms. Meanwhile, the deprivation of total BCAA caused severe immune and metabolic defects. Furthermore, almost no significant linear regression relationship between dietary levels and each physiological change was observed.

Mice without BCAA intake kept losing weight during the whole group, showing the importance of BCAAs in body development. Meanwhile, the feed intake in the 0BCAA group was as much as the other groups, suggesting their collapsed energy metabolism. The nutritional and metabolic block forced the undernourished mice to break down fats and muscle to meet the energy requirement, which caused the relative decrease in muscle weight, WAT weight, and serum glucose levels, and increased serum FFA. The decreased relative spleen weight after BCAA deprivation may be the main sign of health problems. Transcriptome analysis also indicated that the diet without BCAA triggered marked suppression of the immune system. The present results are not surprising because the deficiency of essential amino acids results in impaired metabolism and immune function [14]. However, researchers paid little attention to the impact of BCAAs on immune resistance. When dealing with diabetes or obesity through dietary BCAA intervention, the immune affections of BCAAs should be considered together.

Lipid accumulation is an obvious sign of obesity development, which is also the direct reflection of BCAAs affecting lipid metabolism. Previous studies demonstrated that BCAA supplementation maintained muscle mass and inhibited obesity development in mice [7,15,16,17]. However, it has been reported that reducing BCAA consumption decreased WAT and muscle mass [11,12]. No changes in body weight and body composition were observed when intervening with leucine [18]. We observed that 1/2BCAA and 2BCAA dietary levels both reduced relative WAT weight and increased relative BAT weight in lean mice. In addition, the reduced muscle percentage in the 1/2BCAA group confirmed that BCAA promotes muscle development and suppresses muscle loss [19]. Leptin has an anti-obesity effect that decreases adiposity but maintains muscle mass [20,21]. Serum leptin levels elevated by 1/2BCAA and 2BCAA diets were perhaps a reason for the WAT loss. In addition, BAT is a thermogenic organ that enhances fat consumption and regulates BCAA catabolism [22]. Meanwhile, branched-chain aminotransferase (*Bcat2*), the rate-limiting BCAA catabolic enzyme, was up-regulated in both 1/2BCAA and 2BCAA groups. The reason could be that both lowering and heightening dietary levels activated BCAA catabolism in healthy mice. Moreover, few genes related to the browning of WAT in lean mice were affected by different dietary BCAA levels.

Insulin resistance is closely related to the incidence of type 2 diabetes. Supplementation of dietary BCAAs could moderately weaken the insulin sensitivity and metabolic efficiency of the mice but cannot induce insulin resistance [23,24]. We found that halving and doubling dietary BCAA levels did not influence glucose homeostasis in healthy lean mice, but high BCAA level showed the facilitation effect of BCAAs on insulin secretion [25]. Complete deprivation of BCAAs leads to hyperinsulinemia, hyperleptinemia, and higher HOMA-IR, which may not mean insulin resistance or leptin resistance, but because of the abnormal health and metabolism status in mice from the 0BCAA group. Still, hyperinsulinemia and hyperleptinemia were undoubtedly involved in the physical degeneration in the 0BCAA group.

Different BCAA levels changed genes associated with lipid and glucose metabolism differently. Fibroblast growth factor 21 (*Fgf21*) promotes lipid metabolism, hepatic gluconeogenesis and insulin sensitivity without affecting hepatic glycogen breakdown [26,27,28]. Additionally, *Fgf21* needs to take effect with enough adipose tissue as an indispensable mediator [29], which explains the low *Fgf21* level in the 0BCAA group. However, *Fgf21* in the 1/2BCAA and 2BCAA groups was not significantly affected, assuming *Fgf21* may not be the most critical signaling pathway in the liver when facing BCAA treatment. Fatty acid synthase (*Fasn*) is a complex multifunctional enzyme that plays an essential role in the synthesis of fatty acids, which was only suppressed in the 2BCAA group, but not the 1/2BCAA group. The unique up-regulation of peroxisome proliferator-activated receptor alpha (*Pparα*), and uncoupling protein 2 (*Ucp2*) that regulates lipid metabolism, proved the positive effects of high BCAA levels. Nevertheless, the increased expression level of the Pparα–Fabp1 axis was always found in DIO mice, and the suppression of the Pparα–Fabp1 axis was considered as a treatment for nonalcoholic steatohepatitis [30], suggesting the potential threat of a high-BCAA diet. Carboxyl ester lipase (*Cel*) and colipase (*Clps*) control the hydrolysis and absorption of lipids, which were down-regulated in DIO mice [31] and up-regulated by increasing dietary BCAA levels in lean mice. Additionally, high dietary BCAA levels stimulated glycogen metabolism by enhancing glycogenesis (glycogen synthase 2, *Gys2* [32]) and glycogenolysis (hexokinase 2, *Hk2* [33]; muscle glycogen phosphorylase, *Pygm* [34]). Intriguingly, though the improvement of half-dietary BCAAs on genes involved in lipid and glucose metabolism is not as significant as that in the 2BCAA group, the 1/2BCAA group significantly ameliorated insulin sensitivity. The loss of cytochrome P450 family 8 subfamily B member 1 (*Cyp8b1*) was proved to improve glucose homeostasis [35], and the up-regulation of *Acat2* could reduce toxic polar lipids and improve insulin sensitivity [36], which may explain the better glucose homeostasis in many studies when reducing dietary BCAA levels. Taken together, the difference in gene expression between the 1/2BCAA and 2BCAA groups may be the key to clarifying the mechanism when using dietary BCAA intervention. These differences may be enlarged after four weeks and perhaps reverse their advantages in metabolism, which warrants further studies.

In summary, complete deprivation of BCAAs resulted in metabolic disorders and finally caused the development of weight loss, hyperinsulinemia, and hyperleptinemia in lean mice within four weeks. Increasing and decreasing dietary BCAA levels reduced the body fat rate, but only maintained muscle mass in mice through the high BCAA diet. Further, doubled and half dietary BCAA levels improved metabolic status via regulating lipid and glucose metabolism-related genes. The differences between the 1/2BCAA and 2BCAA groups may provide references for the controversy about BCAA intervention. At least in the present study, low and high BCAA levels in the diet achieved favorable effects on metabolic health in lean mice before the fourth week. Further research is needed to determine the impact of single BCAA removal or long-term BCAA regulation.

## 4. Materials and Methods

### 4.1. Animals and Diets

The study was approved by Northeast Agricultural University Animal Science Research Ethics Committee (NEAU-[2011]-9) (Approval Code: NEAUEC20200202; Approval Date: 3 April 2020). All mice were purchased from HFK Biotechnology Co., Ltd. (Beijing, China) and housed in a temperature-controlled (22 ± 2 °C) and humidity-controlled (55 ± 5%) environment, on a 12 h light/dark cycle with free access to food and water. Before the beginning of the experiment, a total of 60 eight-week-old male C57BL/6J mice were acclimated to a control diet (amino acid-customized diet) for 7 days and then randomly divided into following diets for 4 weeks: 0BCAA (without BCAA), 1/2BCAA (half the normal BCAA level), 1BCAA (normal BCAA level, control group), and 2BCAA (twice the normal BCAA level). Mice selected for each group have a similar average initial body weight. Body weight and food intake were recorded weekly. To formulate isonitrogenous and isoenergetic diets, and minimize the interference of other amino acids, dietary nitrogen was balanced by proportionally supplementing amino acid mixtures, except for BCAAs (Table 2).

### 4.2. Glucose Tolerance Test (GTT) and Insulin Tolerance Test (ITT)

In the third week, the glucose tolerance test (GTT) and insulin tolerance test (ITT) were performed following an intraperitoneal glucose (2 g/kg) and insulin injections (0.75 units/kg) after 12 h or 6 h starvation of the mice, respectively. Blood samples were collected from the tail vein at 0, 15, 30, 60 and 120 min after the injection, and glucose levels were measured with a glucose meter (Roche Diagnostics, Shanghai, China). The fasting serum glucose levels were determined by a colorimetric assay (Nanjing Jiancheng Bioengineering Institute, Nanjing, China), and insulin levels were assessed using a commercial ELISA kit (Nanjing Jiancheng Bioengineering Institute, Nanjing, China), according to the manufacturer’s instructions. The status of insulin resistance was applied by a homeostasis model assessment of insulin resistance (HOMA-IR) and the improved insulin sensitivity index (ISI). The HOMA-IR index was calculated using the following formula: HOMA-IR = [fasting glucose levels (mmol/L)] × [fasting serum insulin (mU/L)]/22.5. The ISI × 100 index was calculated using the following formula: ISI × 100 = 1/[fasting glucose levels (mmol/L)] × [fasting serum insulin (mU/L)] × 100.

### 4.3. Sample Collection

At the end of the experiment, the mice were deprived of food overnight. Blood samples were obtained from the orbital vein in mice anesthetized with ether. Blood was centrifuged at 3000× *g* for 15 min at 4 °C, to collect serum that was stored at −80 °C until use. The mice were sacrificed by neck breaking. The liver, spleen, kidney, white adipose tissue (WAT), brown adipose tissue (BAT), and gastrocnemius muscle were quickly removed, weighed and immediately frozen in liquid nitrogen and stored at −80 °C until analysis.

### 4.4. Histological Analysis

Histological analysis of the liver in mice was carried out with the H&E staining method [9]. The liver fragments fixed in 4% paraformaldehyde buffer were dehydrated and embedded in paraffin to produce random 6 μm thick cuts and stained with hematoxylin and eosin for visualization through an Olympus microscope (Tokyo, Japan).

### 4.5. Serum Parameter Determination

Serum levels of triglycerides (TG), total cholesterol (TC), high-density lipoprotein cholesterol (HDL-C), low-density lipoprotein cholesterol (LDL-C), glucose, free fatty acids (FFA), and blood urea nitrogen (BUN) were determined by enzymatic methods, using commercial diagnostics kits (Nanjing Jiancheng Bioengineering Institute, Nanjing, China). The adiponectin (ADPN) and leptin (LEP) levels were assessed using a commercial ELISA kit (Nanjing Jiancheng Bioengineering Institute, Nanjing, China).

### 4.6. Transcriptomic Analysis and Data Processing

The total RNA samples isolated from experimental groups were subjected to gene expression analysis. The Qubit^®^RNA Assay Kit in Qubit^®^2.0 Flurometer (Life Technologies, Carlsbad, CA, USA) was used to measure the RNA concentration, the RNA Nano 6000Assay Kit of the Bioanalyzer 2100 system (Agilent Technologies, Santa Clara, CA, USA) was used to assess RNA integrity. The sequencing libraries were generated using NEBNext^®^UltraTMRNA Library Prep Kit for Illumina^®^ (NEB, Ipswich, MA, USA), following the manufacturer’s recommendations, and index codes were added to attribute the sequences to each sample. Then, mRNA was purified, and its purity was re-assessed using poly-oligo-attached magnetic beads. A stringent significance threshold (*p*-value < 0.05) was used to limit the false-positive findings, and the fold change ≥1 was chosen to screen a manageable number of genes. The Gene Ontology (GO) Database was used to detect the significant function of differentially expressed genes (DEG) from the level of biological process (BP), cellular component (CC) and molecular function (MF). The canonical pathways of DEGs were analyzed using the Kyoto Encyclopedia of Genes and Genomes (KEGG) database.

### 4.7. Quantitative Real-Time PCR Analysis

The total RNA of liver tissue samples was extracted using the Trizol reagent (TaKaRa, Dalian, China). Before cDNA synthesis, the purity and concentration of RNA were evaluated by absorbance at 260/280 nm. The cDNA was obtained by reverse transcription using PrimeScriptTM RT kit (Takara, Dalian, China). Quantitative real-time PCR was performed using an SYBR Premix Ex TaqTM Kit (Takara, Dalian, China) on an ABI 7500 Fast Real-Time PCR System (Applied Biosystems, Bedford, MA, USA). Calculations were performed using a comparative method (2^−ΔΔCt^), and *β-actin* was used as the internal control. The sequences of primers for the PCR are shown in Table 3.

### 4.8. Western Blot Analysis

The tissues were homogenized at 4 °C in RIPA lysis buffer with a 1% protease inhibitor cocktail, as described previously [9]. The tissue homogenates were centrifuged (12,000× *g*, 15 min, 4 °C), and a Pierce BCA Protein Assay Kit (Thermo Scientific, Waltham, MA, USA) was used to determine the total protein concentration of lysate. Equal amounts of proteins were diluted with the loading buffer and heated in boiling water for 5 min. The protein sample was separated on a 12% SDS−polyacrylamide gel electrophoresis and transferred to PVDF membranes overnight. After blocking with 3% BSA in Tris−Tween buffered saline for 2 h at room temperature, the membranes were incubated with the following antibodies overnight at 4 °C: fatty acid synthase (FAS) (1:1000, Absin) and fibroblast growth factor 21 (FGF21) (1:1000, Absin). The membranes were washed with TBST three times and incubated for 3 h with the peroxidase-conjugated secondary antibody (1:2000, Cell Signaling). The band was visualized by ECL detection systems (Thermo Scientific, Waltham, MA, USA). The images were detected on a Fujifilm LAS-3000 (Tokyo, Japan). The Image J software 1.80 (National Institutes of Health, Bethesda, MD, USA) was used to quantify the protein densitometry.

### 4.9. Statistical Analysis

Data were analyzed with the one-way analysis of variance (ANOVA), using the general linear model procedure in SPSS 26.0 (IBM SPSS Statistics, Chicago, IL, USA, 2019), Image J 1.80 (National Institutes of Health, Bethesda, MD, USA, 2021), and GraphPad Prism 8.3.0 (GraphPad Software, La Jolla, CA, USA, 2012). For multiple comparisons, a Tukey’s multiple comparison test, LSD multiple-comparison test, and Duncan’s multiple comparison test were used. The results are presented as the mean and standard error of the mean for the effect of protein restriction. Differences were considered significantly different at *p* < 0.05, with a trend toward significance at *p* < 0.10.

## Figures and Tables

**Table 1:** Selected significant differentially expressed genes from transcriptomic analysis.

ID	log2FoldChange	*p*-Value	Direction	Gene Name
***0 BCAA* vs. *1 BCAA***				
ENSMUSG00000030827	−1.4493	0.002	Down	*Fgf21*
ENSMUSG00000054422	−1.5147	0.026	Down	*Fabp1*
ENSMUSG00000027533	−0.9817	0.011	Down	*Fabp5*
ENSMUSG00000028240	1.4359	0.019	Up	*Cyp7a1*
ENSMUSG00000050445	−2.2162	<0.001	Down	*Cyp8b1*
ENSMUSG00000000440	−0.5740	0.030	Down	*Pparg*
ENSMUSG00000022383	−0.6585	0.070	Down	*Pparα*
ENSMUSG00000033685	−0.8187	0.008	Down	*Ucp2*
ENSMUSG00000000628	−1.5350	0.014	Down	*Hk2*
***0.5 BCAA* vs. *1 BCAA***				
ENSMUSG00000027533	1.5888	0.003	Up	*Fabp5*
ENSMUSG00000003123	0.5969	0.006	Up	*Hsl*
ENSMUSG00000028240	−0.8180	<0.001	Down	*Cyp7a1*
ENSMUSG00000000440	−0.6348	0.011	Down	*Pparg*
ENSMUSG00000033685	−0.4057	0.052	Down	*Ucp2*
ENSMUSG00000024225	11.4522	0.003	Up	*Clps*
ENSMUSG00000026818	12.3784	0.002	Up	*Cel*
ENSMUSG00000032648	1.4813	0.031	Up	*Pygm*
ENSMUSG00000023832	1.8763	0.005	Up	*Acat2*
ENSMUSG00000030826	0.5064	0.008	Up	*Bcat2*
***2 BCAA* vs. *1 BCAA***				
ENSMUSG00000027533	1.4697	0.026	Up	*Fabp5*
ENSMUSG00000003123	0.8616	<0.001	Up	*Hsl*
ENSMUSG00000024225	26.1467	<0.001	Up	*Clps*
ENSMUSG00000050445	−0.6652	0.031	Down	*Cyp8b1*
ENSMUSG00000022383	0.4712	0.062	Up	*Pparα*
ENSMUSG00000026818	27.1306	<0.001	Up	*Cel*
ENSMUSG00000032648	2.6947	<0.001	Up	*Pygm*
ENSMUSG00000030244	1.3039	0.010	Up	*Gys2*
ENSMUSG00000000628	1.2138	0.030	Up	*Hk2*
ENSMUSG00000030826	0.3879	0.063	Up	*Bcat2*
***0.5 BCAA* vs. *2 BCAA***				
ENSMUSG00000030244	−0.9722	0.087	Down	*Gys2*
ENSMUSG00000023832	1.7224	0.010	Up	*Acat2*
ENSMUSG00000033685	−0.6000	0.020	Down	*Ucp2*
ENSMUSG00000028240	−0.9493	0.052	Down	*Cyp7a1*

F: Forward primer; R: Reverse primer; *Fgf21*: Fibroblast growth factor 21; *Fasn*: Fatty acid synthase; *Hsl*: Hormone sensitive lipase; *Fabp1*: Fatty acid-binding protein 1; *Fabp5*: Fatty acid-binding protein 5; *Cel*: Carboxyl ester lipase; *Clps*: Colipase; *Cyp7a1*: Cytochrome P450 family 7 subfamily A member 1; *Cyp8b1*: Cytochrome P450 family 8 subfamily B member 1; *Pparg*: Peroxisome proliferator-activated receptor gamma; *Pparα*: Peroxisome proliferator-activated receptor alpha; *Ucp2*: Uncoupling protein 2; *Bcat2*: Branched-chain aminotransferase 2; *Acat2*: Acetyl-Coenzyme A acetyltransferase 2; *Hk2*: Hexokinase 2; *Gys2*: Glycogen synthase 2; *Pygm*: Muscle glycogen phosphorylase.

**Table 2:** The composition of diets. 1BCAA (fully purified amino acid-specified diet), 0BCAA (without BCAA), 1/2BCAA (with half the BCAA level in 1BCAA), and 2BCAA (with twice the BCAA level in 1BCAA) for 4 weeks.

Amino Acid Defined Diets	0BCAA	1/2BCAA	1BCAA	2BCAA
Ingredient composition, g/kg as fed				
L-Alanine	5.7	5.1	4.6	3.5
L-Arginine HCl	9.5	8.6	7.7	5.9
L-Aspartic acid	15.0	13.6	12.2	9.4
L-Cystine	4.6	4.1	3.7	2.8
L-Glutamic acid	44.7	40.5	36.3	27.9
Glycine	3.9	3.6	3.2	2.5
L-Histidine HCl, monohydrate	5.7	5.1	4.6	3.5
L-Lysine HCl	16.0	14.5	13.0	10.0
L-Methionine	5.7	5.1	4.6	3.5
L-Proline	25.2	22.9	20.5	15.8
L-Serine	11.9	10.8	9.7	7.5
L-Threonine	8.2	7.5	6.7	5.2
L-Tryptophan	2.6	2.3	2.1	1.6
L-Tyrosine	11.5	10.4	9.3	7.1
L-Phenylalanine	10.8	9.8	8.8	6.8
L-Isoleucine	-	4.3	8.6	17.2
L-Leucine	-	7.7	15.4	30.8
L-Valine	-	5.0	10.0	20.0
Sucrose	304.5	304.5	304.5	304.5
Corn Starch	302	302	302	302
Maltodextrin	75	75	75	75
Vitamin Mix	10	10	10	10
Mineral Mix, AIN-93	35	35	35	35
Choline Bitartrate	2.5	2.5	2.5	2.5
Corn oil	40	40	40	40
Cellulose	50	50	50	50
% kcal from				
Protein	18.8	18.8	18.8	18.8
Carbohydrates	71.8	71.8	71.8	71.8
Fat	9.4	9.4	9.4	9.4
kcal/g	3.8	3.8	3.8	3.8

**Table 3:** Primer sequence for RT-qPCR.

Genes	GenBank ID	Forward Sequence	Reverse Sequence
*β-Actin*	XM_021187106.2	CAGGCATTGCTGACAGGATG	TGCTGATCCACATCTGCTGG
*Fgf21*	NM_020013.4	GTGTCAAAGCCTCTAGGTTTCTT	GGTACACATTGTAACCGTCCTC
*Fasn*	NM_001146708.1	TGCTTGCTGGCTCACAGTTA	ATCAGTTTCACGAACCCGCC
*Hsl*	XM_029479277.1	TGGTGACACTCGCAGAAGAC	GATGGCAGGTGTGAACTGGA
*Fabp1*	NM_017399.5	GTACCAATTGCAGAGCCAGG	CATGGTCTCCAGTTCGCACT
*Fabp5*	NM_001272097.1	TGAAAGAGCTAGGAGTAGGACTG	CTCTCGGTTTTGACCGTGATG
*Cel*	NM_009885.2	AGGACAACACCTATGGGCAAG	CTCCTCCCCGTCATACAGGTA
*Clps*	NM_001317065.1	GAACAGTATGCAGTGTAAGAGCA	GCAGATGCCATAGTTGGTGTTG
*Cyp7a1*	NM_007824.3	AAACTCCCTGTCATACCACAAAG	TTTCCATCACTTGGGTCTATGC
*Cyp8b1*	NM_010012.3	TGTACAGTGCTAGGAGCCCT	GCTCAGGAAGCCAGCCTTAA
*Pparg*	NM_133249.3	TCCTGTAAAAGCCCGGAGTAT	GCTCTGGTAGGGGCAGTGA
*Pparα*	NM_001113418.1	CCATCTGTCCTCTCTCCCCA	TTGCAGCTCCGATCACACTT
*Ucp2*	NM_011671.5	ATGGTTGGTTTCAAGGCCACA	CGGTATCCAGAGGGAAAGTGAT
*Bcat2*	NM_001243052.2	CAGCCACACTAGGACAGGTCT	CAGCCTTGTTATTCCACTCCAC
*Acat2*	NM_009338.3	CCCGTGGTCATCGTCTCAG	GGACAGGGCACCATTGAAGG
*Hk2*	NM_013820.3	TGATCGCCTGCTTATTCACGG	AACCGCCTAGAAATCTCCAGA
*Gys2*	NM_145572.2	GAGTGGGGAGAGAATTACTTCCT	GGGCTCACATTGTTCTACTTGA
*Pygm*	NM_011224.2	CTTAGCCGGAGTGGAAAATGT	GTAATCTCTCGGAGTAGCCACA

F: Forward primer; R: Reverse primer; *Fgf21*: Fibroblast growth factor 21; *Fasn*: Fatty acid synthase; *Hsl*: Hormone sensitive lipase; *Fabp1*: Fatty acid-binding protein 1; *Fabp5*: Fatty acid-binding protein 5; *Cel*: Carboxyl ester lipase; *Clps*: Colipase; *Cyp7a1*: Cytochrome P450 family 7 subfamily A member 1; *Cyp8b1*: Cytochrome P450 family 8 subfamily B member 1; *Pparg*: Peroxisome proliferator-activated receptor gamma; *Pparα*: Peroxisome proliferator-activated receptor alpha; *Ucp2*: Uncoupling protein 2; *Bcat2*: Branched-chain aminotransferase 2; *Acat2*: Acetyl-Coenzyme A acetyltransferase 2; *Hk2*: Hexokinase 2; *Gys2*: Glycogen synthase 2; *Pygm*: Muscle glycogen phosphorylase.

## Data Availability

Not applicable.

## Supplementary Materials

The following supporting information can be downloaded at: https://www.mdpi.com/article/10.3390/ijms24065401/s1.
