# Supplementary material for: Short-Term Decreasing and Increasing Dietary BCAA Have Similar, but Not Identical Effects on Lipid and Glucose Metabolism in Lean Mice
Source: Int J Mol Sci. 2023 Mar 11;24(6):5401. doi: 10.3390/ijms24065401 (PMC10049642; doi:10.3390/ijms24065401)
Supplement: Supplementary file 1 [file ijms-24-05401-s001.zip › ijms-2120707-supplementary.docx]

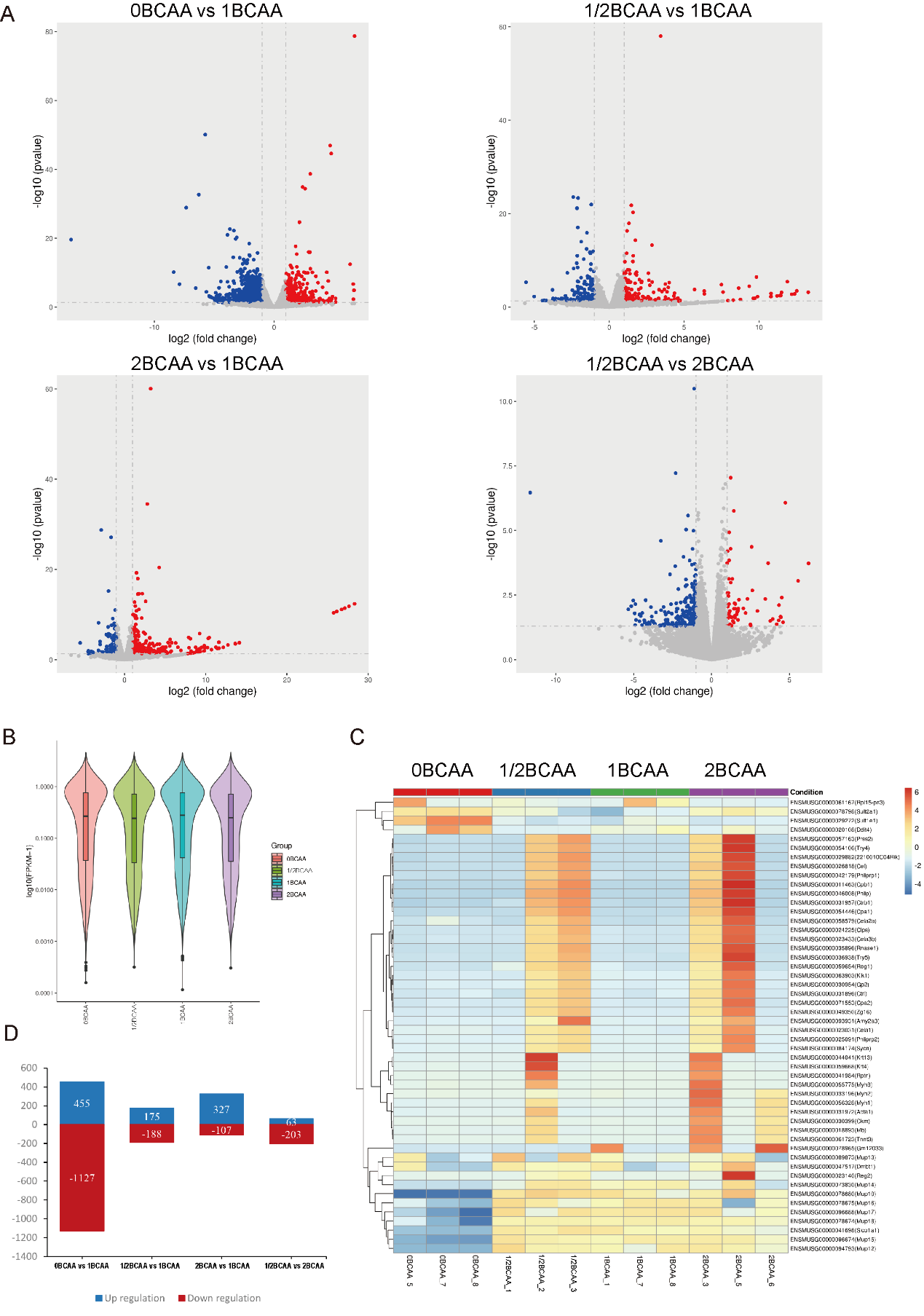


**Figure S1**. Transcriptomic analysis of the effects of different dietary BCAA levels on lean mice (n = 3). (**A**): Volcano plot showing differentially expressed genes in the liver of mice with different dietary BCAA treatments; (**B**): Violin plot of gene expression density estimation in mice; (**C**): Top 50 differentially expressed genes among four groups; (**D**): Numbers of differentially up-regulated and down-regulated differentially expressed genes.
